# Awareness of difficult airway equipment on the ICU

**DOI:** 10.1186/cc11132

**Published:** 2012-03-20

**Authors:** A Wozniak, A Iyer

**Affiliations:** 1 Royal Liverpool University Hospital, Liverpool, UK

## Introduction

It is widely recognised that critically ill patients can be difficult to intubate, requiring the use of advanced airway skills and equipment.The range of airway equipment necessary for patients on the ICU has recently been recommended [1]. Our ICU has a comprehensive difficult airway trolley (DAT) which is regularly maintained. With a high turnover of trainees, we were keen to determine if there was a training need to be met regarding airway management in ICU patients. The objectives were to determine awareness of the DAT, assess knowledge of its contents and ascertain confidence in its use.

## Methods

We audited against previously described standards [1] using a short questionnaire, disseminated to trainees and consultants working on the ICU in November 2010: 100% of clinicians should be aware of the location and contents of the DAT; 100% of anaesthetists should have had difficult airway equipment training. A re-audit was conducted in June 2011 to complete the audit cycle.

## Results

One hundred per cent of clinicians were aware of the DAT. Only 35% had read the folder detailing its contents with instructions. Ninety per cent could confidently name the equipment which should be readily available for difficult intubations but only 70% were confident to use it unaided. Fifty per cent would request the presence of an operating department practitioner (ODP) for an unplanned intubation on the ICU. Twenty-eight per cent were not airway trained. Re-audit showed 100% of respondents were aware of the equipment. Sixty per cent had confidence in its use, a similar proportion to the original audit. Eighty per cent would have an ODP for unplanned intubations. One hundred per cent were airway trained. *Outcomes *A designated consultant was assigned to teach difficult airway management at quarterly departmental induction sessions which included equipment location and algorithms. Trainees and consultants underwent simulation and mannequin training, including tracheostomy and surgical airway management. Regular updates and case-based teaching sessions were implemented. Airway proficiency assessments were conducted at induction.

## Conclusion

This audit highlights our variable workforce. The presence of junior, nonairway-trained staff on the ICU calls for regular, compulsory airway teaching sessions for all, regardless of grade. Airway competency must be formally assessed at the start of an ICU attachment. Airway instructions for challenging patients should be clearly documented with advice on access to senior assistance for emergencies.

## References

[B1] Jeanrenaud P Difficult airway trolleys for the critical care unit JICS 2010 11 98 103

